# Cardiac evaluation of young athletes: Time for a risk‐based approach?

**DOI:** 10.1002/clc.23364

**Published:** 2020-04-03

**Authors:** Hamish MacLachlan, Jonathan A. Drezner

**Affiliations:** ^1^ Cardiovascular Sciences Research Centre St Georges University of London London UK; ^2^ Department of Family Medicine and the Center for Sports Cardiology University of Washington Seattle Washington USA

**Keywords:** athlete, ECG, risk, screening, sports cardiology, sudden cardiac death

## Abstract

Pre‐participation cardiovascular screening (PPCS) is recommended by several scientific and sporting organizations on the premise that early detection of cardiac disease provides a platform for individualized risk assessment and management; which has been proven to lower mortality rates for certain conditions associated with sudden cardiac arrest (SCA) and sudden cardiac death (SCD). What constitutes the most effective strategy for PPCS of young athletes remains a topic of considerable debate. The addition of the electrocardiogram (ECG) to the medical history and physical examination undoubtedly enhances early detection of disease, which meets the primary objective of PPCS. The benefit of enhanced sensitivity must be carefully balanced against the risk of potential harm through increased false‐positive findings, costly downstream investigations, and unnecessary restriction/disqualification from competitive sports. To mitigate this risk, it is essential that ECG‐based PPCS programs are implemented by institutions with a strong infrastructure and by physicians appropriately trained in modern ECG standards with adequate cardiology resources to guide downstream investigations. While PPCS is compulsory for most competitive athletes, the current debate surrounding ECG‐based programs exists in a binary form; whereby ECG screening is mandated for all competitive athletes or none at all. This polarized approach fails to consider individualized patient risk and the available sports cardiology resources. The limitations of a uniform approach are highlighted by evolving data, which suggest that athletes display a differential risk profile for SCA/SCD, which is influenced by age, sex, ethnicity, sporting discipline, and standard of play. Evaluation of the etiology of SCA/SCD within high‐risk populations reveals a disproportionately higher prevalence of ECG‐detectable conditions. Selective ECG screening using a risk‐based approach may, therefore, offer a more cost‐effective and feasible approach to PPCS in the setting of limited sports cardiology resources, although this approach is not without important ethical considerations.

## 1 INTRODUCTION

The sudden death of a young athlete is a devastating event, particularly when one considers its unexpected nature and the considerable number of life‐years lost for an individual who is deemed to represent the healthiest segment of our society. As such, these highly emotional events are afforded significant visibility and galvanize discussion between physicians and the lay community with an emphasis on improving our understanding of the conditions predisposing to sudden cardiac arrest (SCA)/sudden cardiac death (SCD) and the development of effective preventative strategies.

Exercise is a recognized trigger for ominous ventricular tachyarrhythmias in predisposed athletes that harbor a hereditary or congenital cardiac abnormality associated with SCA/SCD. Naturally, there is a desire to identify these at‐risk conditions on the premise that the majority of these athletes can be detected during life through pre‐participation cardiovascular screening (PPCS). The primary objective of PPCS is to identify underlying cardiac disorders predisposing to SCA/SCD with the intent to reduce morbidity and mortality by mitigating risk through individualized, patient‐centered, and disease‐specific medical management.^1^ There is widespread agreement that SCA/SCD in young athletes is an important public health issue, and that effective prevention requires early detection of these cardiac conditions. Compelling evidence for its justification has led the American Heart Association (AHA) and the European Society of Cardiology (ESC) to both advocate PPCS for young athletes on medical, legal, and ethical grounds.

Determining what constitutes the most effective PPCS strategy for young athletes has created an intense debate regarding the need and feasibility of electrocardiogram (ECG)‐based screening programs. Critics of a widespread ECG‐based screening program highlight concerns related to the lack of robust evidence for its efficacy to reduce athlete fatalities, reliability of outcomes (false‐positives), and overall cost. Proponents of ECG screening recognize the relative failure of the history and physical examination to meet the primary objective of PPCS, specifically to detect athletes with at‐risk cardiac conditions, and the wealth of evidence demonstrating the high false‐positive response rate and very low positive predictive value of symptom and family history questionnaires.^2^

The paradigm of ECG screening has been debated in a binary “all or nothing” form, whereby programs are mandated to include ECG for all young athletes, or none at all. These polarized options contradict the fundamental approach to preventative medicine, which ordinarily requires assessment of individualized patient risk and the available medical resources. This article will address the current landscape of PPCS and review the epidemiological data of SCA/SCD in young athletes, which may support a novel risk‐based approach to PPCS.

## 2 ETIOLOGY OF SCA/SCD IN YOUNG ATHLETES

Understanding the etiology of SCA/SCD is paramount to inform the development of an effective preventative strategy for young athletes. Hypertrophic cardiomyopathy (HCM) is historically recognized as the leading cause of SCA/SCD in the United States while in Italy, arrhythmogenic cardiomyopathy predominates. Genetic variation, ascertainment bias of identified cases, and variable criteria and expertise for pathological diagnosis contribute to these regional discrepancies. More recent data suggest that autopsy negative sudden unexplained death in athletes with presumed SCD may be more prevalent than previously thought. Data from a specialist cardiac pathology center in the United Kingdom in 357 athletes has shown that in up to 42% of cases, the heart is structurally normal, and when the toxicology screen is negative, these deaths are classified as sudden arrhythmic death syndrome (SADS).^3^ This finding also has been demonstrated in studies of college athletes in the United States,^4^ military recruits,^5^ and the general population (nonathletes).^6^ These cases are largely attributed to primary cardiac ion channel disorders such as the Brugada syndrome, long QT syndrome, and catecholaminergic polymorphic ventricular tachycardia, or congenital accessory pathways such as ventricular preexcitation. Accurate diagnosis following SADS, enhanced by postmortem genetic testing and standardized autopsies performed by experienced cardiac histopathologists, is essential when we consider that subsequent evaluation of SADS families leads to a diagnosis of an inherited cardiac condition in up to 50% of cases.^7^

## 3 INCIDENCE OF SCA/SCD IN YOUNG ATHLETES

SCA/SCD is the leading medical cause of death in young athletes during sports and exercise.^4^ Current estimates for the incidence of SCA/SCD in young athletes vary widely. This variation is accounted for by differing methodology and heterogeneous population comparisons. An accurate calculation of the incidence of SCA/SCD requires a precise numerator (number of cardiac events per year) and an exact denominator (number of athlete participants per year) in the population studied. Inaccurate assessment in either of these accounts for unreliable estimates of incidence. The majority of studies have utilized passive collection methods through retrospective review of media reports, electronic databases, and insurance claims, which are limited by ascertainment and confirmation bias that may significantly underestimate incidence calculations. Mandatory reporting systems of athlete deaths with accurate population demographics offer the most reliable method of case identification and incidence calculations, although very few currently exist.

Survival rates of SCA in athletes have significantly improved following more widespread implementation of emergency response plans and automated external defibrillators (AEDs).^8^, ^9^ It is therefore essential that both nonsurvivors (SCD) and survivors (SCA) are included in estimations of incidence. Studies failing to do so provide a worrying misconception of declining rates of SCD where the actual rate of life‐threatening cardiac events is unchanged and the purpose of identifying athletes with at‐risk disorders through PPCS remains of critical importance. Other methodological factors, which influence incidence estimates include the definition of an “athlete,” the inclusion or exclusion of adverse events at certain times or locations (some studies include events which occur only during exercise), and finally the age range of the study population.

Appreciation of these methodological inconsistencies is particularly important when scrutinizing the validity of estimates drawn from larger systematic reviews. Mohaneney et al recently evaluated the global incidence of sports‐related SCD in young athletes through a meta‐analysis of 21 studies which included 1994 cases of sports‐related SCD over 430 million athlete‐years (AY). The pooled incidence of SCD reported was 0.72 per 100 000 AY. However, the significant variation of reported incidence (0.09‐13.09 per 100 000 AY) across the 21 studies is attributable to considerable heterogeneity in study methodology as described above, and evenly weighting studies with both poor and robust methodology is likely to bias the analysis and underestimate the pooled incidence of SCD.

Harmon et al performed a comprehensive review of studies that have examined the incidence of SCA/SCD in young athletes.^10^ The objective of this review was to assess the methodological strengths and weaknesses used to arrive at estimates, compare studies with estimates of similar populations, and arrive at an approximation of incidence based on the available evidence. The incidence of SCA/SCD across all 28 studies varied from 1:3000 to 1:917 000 AY. However, studies with higher methodological quality yielded a higher incidence ranging from 1:40 000 to 1:80 000 AY.^10^ This systematic review that accounts for differences in study methodology has led to a generally accepted annual incidence of SCA/SCD in young athletes as 1:50 000 AY.

The underpinning of the screening debate is centered on the perceived incidence of SCD in young athletes. The screening community must interpret the validity of incidence estimates with a keen eye on the rigor of the methodology used to obtain them. Further studies using data from mandatory reporting systems and inclusive of all deaths and survivors are clearly warranted if we are to improve our understanding of the magnitude of SCA/SCD in young athletes.

## 4 ARE CERTAIN POPULATIONS OF ATHLETES AT HIGHER RISK OF SCA/SCD?

**FIGURE 1:**
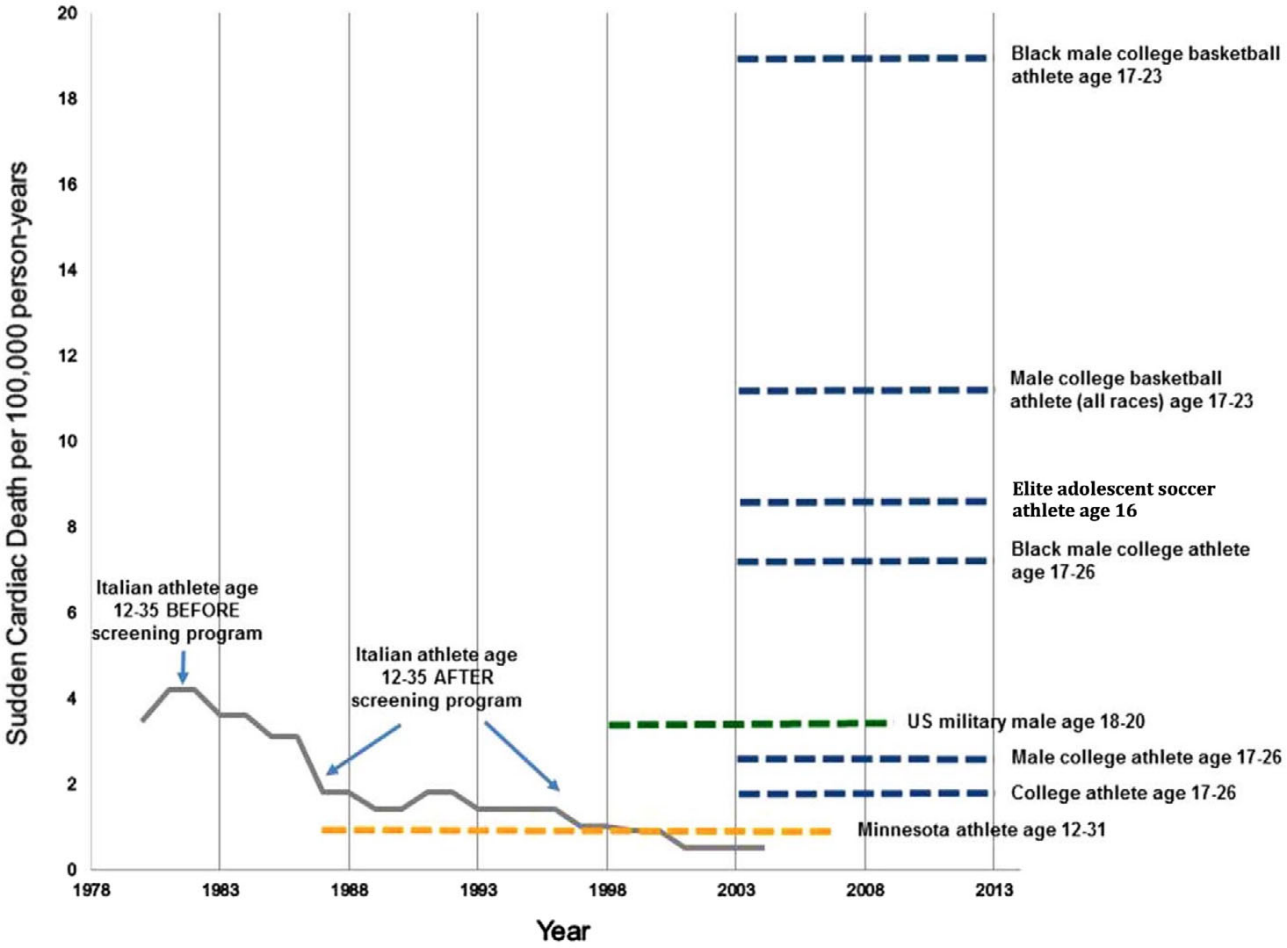
Annual risk of SCD in young athletes.^11^ Annual risk of SCD in athletes from Veneto, Italy,^12^ and Minnesota,^13^ and more recent incidence data in NCAA college athletes,^4^ UK Premier league soccer players,^14^ and US military personnel.^5^ Graph adapted from Drezner et al.^11^ CV, cardiovascular; NCAA, National Collegiate Athletic Association; SCD, sudden cardiac death

Evolving data supports a differential risk profile for SCA/SCD in certain populations of athletes (Figure 1).

**FIGURE 2:**
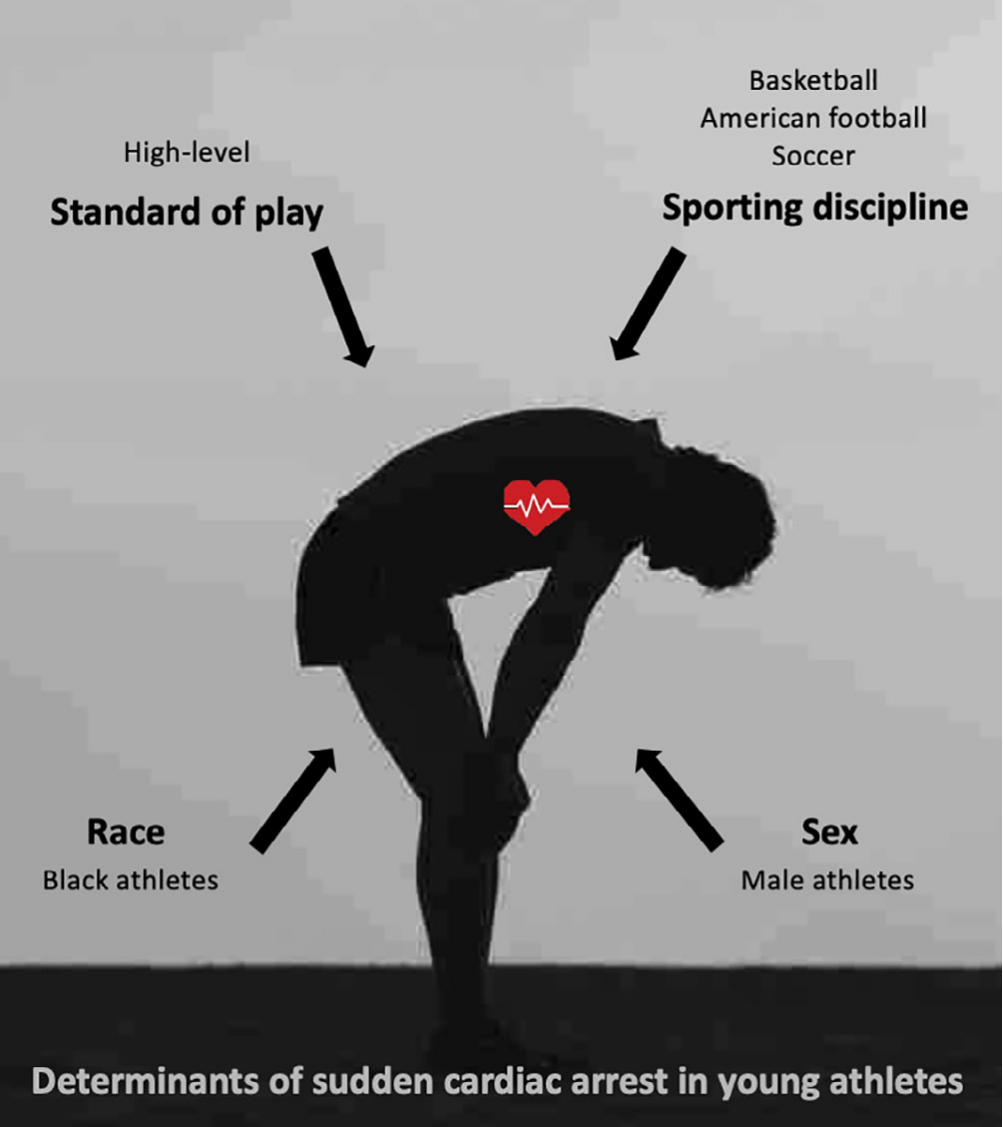
Risk factors for sudden cardiac arrest and death in young athletes

The incidence rate of SCA/SCD in athletes appears to be determined by age, sex, race, sporting discipline, and standard of play (Figure 2). Athletes over 35‐years old are at 5 to 10 times higher risk than their younger counterparts.^10^ Risk in this age group is most commonly attributed to the higher prevalence of ischemic heart disease and established cardiovascular (CV) risk factors (obesity, hypertension, diabetes mellitus, smoking, and hyperlipidaemia). It is well recognized that these CV risk factors at a young age are associated with increased CV morbidity and mortality in later life; but could they promote a more immediate risk of SCA? Jayaraman et al recently evaluated the association of standard CV risk factors and SCA in 3775 young individuals (aged 5‐34 years).^15^ Interestingly, standard CV risk factors were identified in 58% of SCA cases. One might expect this figure to be lower in a selected cohort of young athletes; however, these findings remain relevant to public health policy, and primary preventative approaches which should include educating and treating young athletes with risk factors of CV disease.

Clear sex differences exist, with males reported to be at 3 to 10‐fold higher risk compared to female counterparts in competitive sport, and up to 20‐fold higher risk in recreational sport.^16^ The reasons for this are poorly understood. The modern era has seen a significantly higher proportion of competitive female athletes without a parallel increase in mortality rates. It is thought that these sex differences are determined by a complex interplay between genetic, phenotypic, hormonal, and possibly environmental mechanisms.

Two prominent studies of young athletes in the United States have mirrored these findings with regard to sex, but also suggest that the risk of SCA/SCD is further determined by the athlete's race, sporting discipline, and standard of competition. Garberich et al recently evaluated the demographics of 842 young athletes with autopsy confirmed SCD within a large forensic registry of competitive US athletes over a 32‐year period. The incidence of SCD in male athletes exceeded that in female athletes by 6.5‐fold (1:122 000 vs 1:787 000 AY), while the risk in black athletes exceeded that in white athletes by almost fivefold (1:13 000 vs 1:61 000 AY).^17^

Harmon et al evaluated 79 cardiac‐related deaths taken from a large database of National Collegiate Athletic Association (NCAA) athletes over 10 years which included 4 242 519 total AY of participation. It is worth noting that all athletes had previously undergone PPCS predominantly without an ECG and incidence rates did not include survivors of SCA. The overall incidence of SCD in NCAA athletes was 1:54 000 AY. Again, male athletes were at threefold higher risk than female counterparts (1:38 000 vs 1:12 2000 AY) and black athletes were threefold higher risk than white athletes (1:21 000 vs 1:68 000 AY). Breaking these results down further, male‐black athletes demonstrated a risk of 1:16 000 AY, male basketball players 1:9000 AY, and male NCAA division I basketball players a risk of 1:5200 AY. Men's basketball represents only 4% of male NCAA athletes, but almost 20% of all SCD cases. Male basketball and American‐football players together represent 23% of all male NCAA athletes but 50% of all SCD cases. In combination, these two sports (basketball and American‐football) consistently account for the majority (50%‐61%) of all identified cases of SCA/SCD in the United States.^4^, ^18^ In the United Kingdom, elite adolescent and young adult soccer players also demonstrate a high incidence of SCD (1:14 800 AY).^14^

In a Canadian study, Landry et al undertook a retrospective 5‐year analysis of a population‐based registry of out‐of‐hospital SCA occurring in young athletes.^19^ They identified 16 athletes who had experienced SCA during competitive sport, which suggested a low absolute risk of SCA (0.76 cases per 100 000 or 1:132 187 AY). This figure is largely determined by the extremely low number of SCA cases (0.29 per 100 000 AY) in ice hockey players who accounted for a third of the overall study population (Table 1). Once again, evaluation of incidence according to type of sport suggests that high‐risk groups exist; including those athletes who engage in Jujitsu (21.1 per 100 000 AY), soccer (5.9 per 100 000 AY), and basketball (3.4 per 100 000 AY).^19^ The mechanisms for which these athlete subpopulations are at disproportionately higher risk remains unclear.

**TABLE 1:** Incidence of sudden cardiac arrest among competitive athletes in Ontario and Canada

Sporwt	Percent of total athlete population	SCD, 2009 to 2014	SCD per 100 000 AY	Incidence of SCD (AY)	AY of observation, 2009 to 2014
Jujitsu	0.3	2	27.10	1/3690	7380
Soccer	3.2	4	5.92	1/16 898	67 590
Rugby	1.3	1	3.77	1/26 520	26 520
Basketball	2.7	2	3.45	1/29 004	58 008
Baseball	1.8	1	2.63	1/38 058	38 058
Race events^a^	20.8	4	0.90	1/110 073	440 292
Ice hockey	33	2	0.29	1/349 170	698 340
All sports	100	16	0.76	1/132 187	2 114 994

a Includes endurance events such as triathlons and marathons.

Abbreviations: AY, athlete‐years; SCD, sudden cardiac arrest.

*Source:* Adapted from Landry et al^19^ and D'Silva et al.^20^

## 5 CURRENT PPCS PRACTICE

The AHA recommends that all athletes are screened with a 14‐element assessment via a medical history and a physical examination. Secondary evaluation is considered for any athlete with a positive response to any one of the 14 elements. The AHA's pragmatic approach is widely practiced but limited when one considers that up to 80% of young athletes are asymptomatic prior to their SCA/SCD.^21^, ^22^ Furthermore, the majority of conditions associated with SCA/SCD are seldom associated with abnormal CV findings on physical examination. Indeed, a direct comparison of the performance of the AHA 14‐element evaluation vs ECG in the CV screening of adolescent athletes demonstrated that the sensitivity (18.8%), specificity (68.0%), and positive predictive value (0.3%) of the AHA 14‐point evaluation was substantially lower than the sensitivity (87.5%), specificity (97.5%), and positive predictive value (13.6%) of ECG.^2^ Other studies comparing screening strategies, some of which were undertaken in dedicated centers with PPCS experience, have consistently highlighted the poor performance of the medical history and physical examination when used in isolation. A recent meta‐analysis of 15 studies comparing strategies in 47 137 athletes revealed that the ECG was five times more sensitive than the medical history and 10 times more sensitive than physical examination for detecting athletes with conditions associated with SCA/SCD.^23^

The weight of scientific evidence has led to widespread agreement that ECG enhances the detection of conditions associated with SCA/SCD to better meet the primary objective of PPCS. Screening with ECG may identify more athletes with at‐risk disease, but does this equate to saving lives? Limited long‐term morality data is available to support the efficacy of an ECG‐based PPCS strategy.^12^ Italy introduced a mandatory state sponsored PPCS program in 1982. This program requires all young competitive athletes to undergo assessment with a health questionnaire and a resting 12‐lead ECG prior to clearance for sports participation. Mortality data over a 25‐year period (1979‐2004) demonstrated that the incidence of SCD in young athletes reduced by almost 90%. By contrast, the incidence rate of SCD in unscreened nonathletes remained unchanged over the same time period. Investigators attributed the mortality trends to the greater number of cardiac conditions, specifically cardiomyopathies, identified by an ECG‐based PPCS. This decline in SCD correlated with the number of athletes disqualified from competitive sport, which doubled over the screening period. This seminal study suggests that systematic PPCS of young athletes with ECG significantly reduces mortality rates via identification and disqualification of individuals with previously undiagnosed cardiomyopathies.

Only one other study provides long‐term mortality data in combination with findings from baseline PPCS. Malhotra et al reported findings in 11 168 elite adolescent soccer players screened at a mean age of 16.4 years with a health questionnaire, physical examination, ECG, and echocardiogram, and followed for a mean of 10.6 years. Forty‐two athletes (0.38%) were identified with a cardiac disorder associated with SCA/SCD. Only four of these athletes (9.5%) presented with symptoms and/or findings on physical examination, whereas 36 (86%) had an abnormal ECG. Athletes with pathological cardiac disorders received disease‐specific medical management, procedural interventions, and exercise restrictions as indicated to mitigate their risk. Two athletes with HCM who returned to sport against medical recommendations died, while SCA/SCD was potentially averted in 40 of 42 athletes optimally managed after early detection of a pathological cardiac disorder.^14^ Overall, eight athletes died an average 6.8 years from their screening evaluation. Six of these eight deaths were attributed to cardiomyopathy. One must acknowledge that the ECG performed only once at age 16 failed to detect a critical proportion of athletes who subsequently died from cardiac disease. This limitation may be attributed to the imperfect sensitivity of ECG,^24^ or more likely that cardiac pathology was yet to manifest with ECG anomalies, especially in cases of cardiomyopathy where phenotypic expression of disease in genetically predisposed individuals often occurs in late adolescence and early adulthood.

This raises the important issue regarding the frequency and timing for PPCS when one considers the variable age at which certain conditions manifest on ECG. The optimal age to introduce PPSC for athletes remains largely uncertain. Most consensus guidelines suggest PPCS start at age 12 when pubertal maturation and the expression of many disorders associated with SCD may begin.^1^, ^25^, ^26^ What is more certain, and supported by the findings of Malhotra et al, is that screening should be repeated at regular intervals for the timely identification of phenotype progression. This is reflected in the ESC's recommendation that athletes should undergo regular ECG screening at minimum every 2 years.^25^

While outcome‐based studies remain limited and the natural history of conditions associated with SCA/SCD remains largely unknown, PPCS inclusive of ECG is further supported by disease‐specific data, which demonstrates that early detection in conjunction with individualized risk stratification and management lowers mortality rates for certain cardiac conditions associated with SCA/SCD, including HCM and long QT syndrome.^27^, ^28^ Consequently, the ESC recommends PPCS for all young athletes with the routine inclusion of ECG.

The ESC's uniform approach in favor of ECG screening for all young athletes raises numerous practical limitations, which warrant careful review. The ECG will not detect all conditions, which predispose athletes to SCA/SCD. The ECG may be normal in up to 10% of athletes with HCM, 70% who are genotype positive for long QT syndrome, and 90% with premature coronary artery disease. Furthermore, the resting ECG is normal in almost all individuals with anomalous coronary arteries, catecholaminergic polymorphic ventricular tachycardia, and aortopathies. These false‐negatives may result in false reassurance for a small proportion of athletes that harbor CV conditions associated with SCA/SCD, but the rate of false‐negative screens is substantially lower than if using the less sensitive evaluation of history and physical examination alone.

False‐positive ECG findings are another important limitation to consider. Regular exercise leads to a constellation of electrical and structural cardiac alterations which collectively form the phenotype of the “athlete's heart.” These physiological changes manifest as electrical changes on the athlete's ECG and, in some cases, mimic those observed in patients with cardiomyopathy. A notable example of this is the relatively high prevalence of anterior T‐wave inversion in black male athletes and some adult endurance athletes. Misinterpretation of physiological ECG findings leads to unnecessary downstream investigations and, in some cases, inappropriate restriction from competitive sports. The financial implications of false‐positive findings provide the central argument against national legislation for ECG screening in most countries.

## 6 ECG INTERPRETATION IN ATHLETES

**FIGURE 3:**
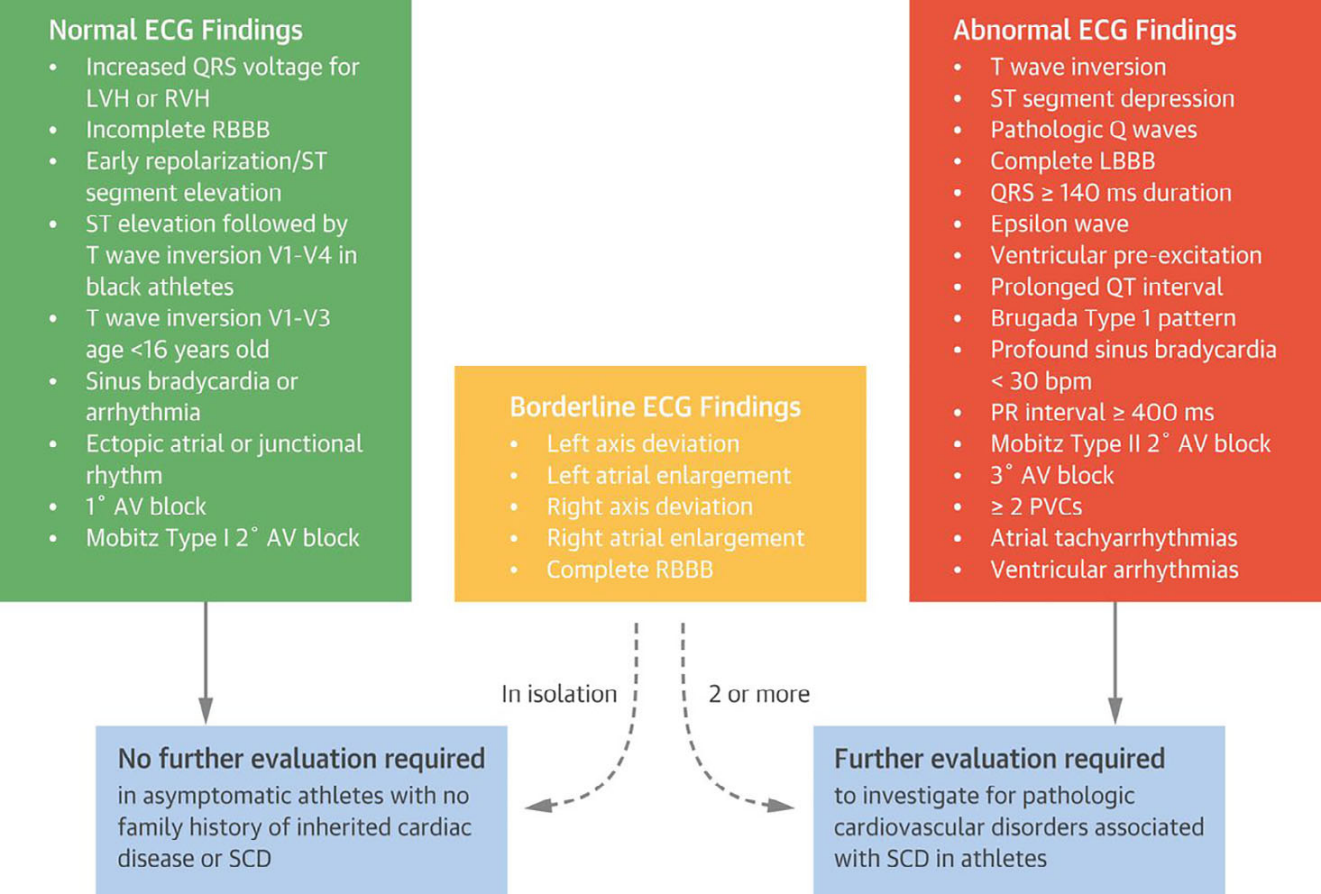
The 2017 International Consensus Standards for electrocardiogram (ECG) interpretation in athletes^29^

Standardized ECG interpretation criteria, first introduced by the ESC in 2010, distinguished physiological adaptations from pathological abnormalities, which led to improvements in interpretation accuracy. Refinement of these criteria over the last decade has been facilitated by a greater understanding of the athlete's heart. Contemporary criteria have increasingly accounted for adolescent athletes, black ethnicity and some nonspecific electrical anomalies, including axis deviation and voltage criteria for atrial enlargement, and sequentially improved the specificity of ECG screening by driving down the false‐positive rate from 25% to less than 5%. In a study of 5258 NCAA athletes, the false‐positive rate was only 1.3% when experienced clinicians applied the latest international criteria (Figure 3) for ECG interpretation in athletes.^30^ Application of contemporary criteria has furthermore been associated with a 27% reduction in the cost of screening without compromising the ability to detect athletes with serious cardiac disease.^31^

Accurate ECG interpretation requires training and creates potential for inter‐observer variation. This limitation was revealed in a recent study, which demonstrated that cardiologists with no experience in PPCS were at least 40% more likely to categorize ECGs as abnormal, compared to those with relevant experience.^32^ Moreover, the inter‐observer agreement rates among experienced cardiologists were only moderate at best. These results highlight the need for increased education in modern standards of ECG interpretation. Online training modules reviewing the international criteria for ECG interpretation in athletes are freely available at: https://uwsportscardiology.org/e-academy/. Efforts to improve the accuracy of ECG interpretation using such methods have proved efficacious in several small studies and hold promise for the future.^33^, ^34^, ^35^

These limitations underpin the need for ECG‐based PPCS programs to be implemented by centers with a strong infrastructure, using high quality control measures and physicians who are appropriately trained in modernized ECG standards and supported by adequate cardiology resources to guide the downstream investigations. Unfortunately, there are very few countries worldwide that can realistically provide such a platform for PPCS, which has driven the PPCS community to consider alternative strategies.

## 7 IS IT TIME FOR A RISK‐BASED APPROACH TO PPCS?

The argument for a risk‐based approach to PPCS is supported by the structure of established screening programs on both sides of the Atlantic. The effectiveness of these programs, including those for abdominal aortic aneurysm (AAA), breast, and colon cancer, is primarily judged by their ability to detect disease, assuming perhaps without definitive evidence that early detection will reduce mortality through modern treatment. Furthermore, these programs consider higher risk groups in their target population. Population‐based screening programs for AAA were first established in the United States and United Kingdom over 10 years ago.^36^, ^37^ The prevalence of AAA increases with age and region, and is four to six times higher in males compared to female counterparts.^38^, ^39^ Epidemiological data, which identifies high‐risk groups is taken into account by national screening committees, considering both programs restrict screening to men over the age of 65 years. Additional risk factors including family history and smoking status are respectively considered for breast cancer and AAA screening programs in the United States.

Guidelines for the primary prevention of CV disease routinely recommend physicians to consider the individual's estimated risk of adverse CV events as a guide to management decisions.^40^ This factor is not considered in the current PPCS recommendations, which adopt a “one size fits all approach,” despite several incidence studies providing robust evidence that certain athletic groups are at higher risk of SCA/SCD than others.

The American Medical Society for Sports Medicine (AMSSM) recently proposed a new PPCS framework, which considers the individual risk of the athlete as well as physician expertise and available cardiology resources for accurate ECG interpretation and the secondary evaluation of ECG abnormalities.^1^ Following careful review of the current evidence and existing knowledge gaps, the task force recommended that physicians should consider more intensive screening strategies, such as ECG screening, for high‐risk athletes.

**FIGURE 4:**
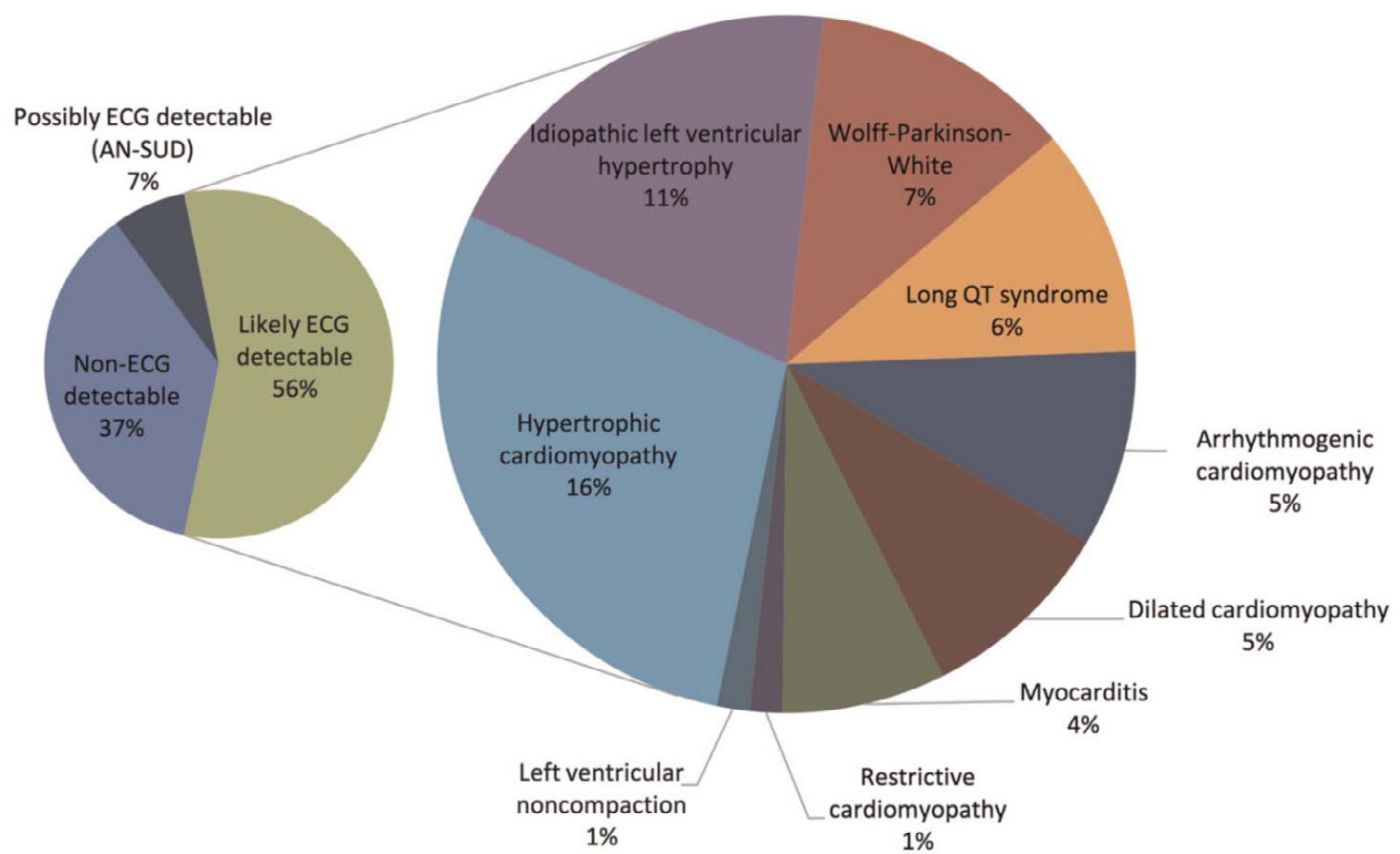
Electrocardiogram‐detectable etiologies implicated in 117 cases of sudden cardiac arrest and death in US competitive athletes (age 11‐29 years)^18^

The AMSSM's risk‐based framework is supported by the outcome of a recent surveillance study of young athletes in the United States. Peterson et al prospectively evaluated the etiology of SCA/SCD cases in the United States through a national surveillance program over a 2‐year period.^18^ Of the 117 cases with a confirmed diagnosis, 66 (56%) were identified with conditions that routinely demonstrate ECG abnormalities (Figure 4). ECG‐detectable conditions were identified in 33 of 62 (54%) white athletes, 23 of 37 (62%) black athletes, and 13 of the 19 (68%) black basketball players. HCM was attributed as the cause of death in 10% of white athletes, 30% of black athletes, 23% of male basketball athletes, and 25% of American‐football athletes. Although this study evaluated a relatively small cohort of athletes, the findings suggest that ECG screening may be most effective in higher risk groups where the proportion of ECG‐detectable conditions is disproportionately higher. Historical studies also highlight HCM as one of the most common causes of SCD in young athletes; a condition which manifests with abnormal ECG findings in up to 90% of individuals.^41^ Larger prospective studies on the etiology of SCA/SCD in athletes are warranted if we are to improve our understanding of what constitutes the most effective PPCS strategy for different athlete risk groups.

When ample sports cardiology resources are available, routine use of ECG is possible in the PPSC of all athletes. However, reserving the ECG for smaller populations of higher risk groups may offer a more pragmatic approach for institutions that are not equipped with the infrastructure and expertise to adequately support an ECG‐based model on a larger scale.

It is prudent, however, to consider the ethical issues of such an approach. Institutions may argue that equivalent screening services should be available to all athletes under their care and not differentiated based on sex, race, or sport. This notion is supported by data from France, Denmark, and the United States, which has demonstrated that the incidence of SCD in recreational athletes and nonathletic individuals, is higher than previously thought.^17^, ^42^, ^43^ While recreational athletes are not required to undergo a PPCS prior to sports participation, should these individuals be precluded from primary preventative strategies? Critics may also argue that a larger number of epidemiological studies, using mandatory reporting systems for case identification, are warranted before we can reliably define “high‐risk” populations. However, nearly all preventive practices in medicine base the rigor of the screening evaluation on the individual risk of the patient, and thus there is justification in the setting of limited resources to provide the most intensive screening to the athletes shown by current evidence to be at highest risk.

## 8 CONCLUSIONS

The paradigm of PPCS has disputed the merits of an ECG‐based program as a binary “all or nothing” approach. This perspective fails to consider the individual risk of the athlete and the available sports cardiology expertise, which are essential to providing sound preventative care. Evolving data supports that certain populations of athletes (male, black, basketball, soccer, or American‐football players) are at higher risk of SCA/SCD than others. The higher prevalence of ECG‐detectable conditions (most notably, cardiomyopathies) reported in these high‐risk groups, favors a more intensive approach with ECG screening. In the setting of limited sports cardiology resources, a risk‐based approach may be the most pragmatic method to perform effective PPCS. While targeted screening for higher risk individuals has precedence in other medical prevention programs, the merits, and feasibility of this strategy must be carefully balanced against the ethical concerns associated with screening only a subset of athletes and the need for more definitive outcomes data.
